# How Does Search for Meaning Lead to Presence of Meaning for Korean Army Soldiers? The Mediating Roles of Leisure Crafting and Gratitude

**DOI:** 10.3389/fpsyg.2021.766798

**Published:** 2022-01-14

**Authors:** Jung In Lim, Jason Yu, Young Woo Sohn

**Affiliations:** Department of Psychology, Yonsei University, Seoul, South Korea

**Keywords:** search for meaning, presence of meaning, leisure crafting, gratitude, Korean military

## Abstract

Many studies demonstrate that finding meaning in life reduces stress and promotes physical and psychological well-being. However, extant literature focuses on meaning in life among the general population (e.g., college students or office workers) in their daily lives. Thus, this study aimed to investigate the mechanisms of how individuals living in life-threatening and stressful situations obtain meaning in life, by investigating the mediating roles of leisure crafting and gratitude. A total of 465 Army soldiers from the Republic of Korea (ROK) participated in two-wave surveys with a 2-week interval. Structural equation modeling analyses indicated that the direct effects between the search for meaning, presence of meaning, leisure crafting, and gratitude were significant, except for the direct relationship between the search for meaning and the presence of meaning, and between leisure crafting and the presence of meaning. We tested indirect effects using a Monte Carlo approach and found that leisure crafting and gratitude sequentially mediated the relationship between the search for meaning and the presence of meaning. Our findings highlight the importance of the motivation behind searching for meaning, the proactive use of leisure time, and gratitude for individuals in stressful situations and controlled lifestyles. Finally, we discuss the implications and limitations of this research and future research directions.

## Introduction

In Korea, adult men (aged 20 years or older) are obligated to serve in the military for 18–22 months, depending on the type of armed forces and duties required. Military service in Korea has several characteristics. First, it is involuntary. Given the unique national security situation of the war not having officially ended, military service is an inevitable duty for Korean men. The involuntary nature of the service is a major stressor for enlisted soldiers. Second, soldiers encounter many risk factors during their military service, such as handling weapons and military equipment, as well as confrontations associated with the military demarcation line. Third, soldiers are required to have a controlled lifestyle, living in all-male groups in restricted spaces and following strict hierarchical orders. Apart from several official trips during which soldiers can leave their posts, their lives during military service are confined within posts, including a separation from intimate social relationships.

Consequently, the experience of military service can constitute a stressful event that causes negative psychological states such as anxiety, stress, and maladjustment (Noh et al., 2016). Therefore, Korean soldiers must identify the factors that lead to experiencing and finding meaning in their lives, thereby lowering their stress and enhancing their mental health in military environments.

We proposed that the search for meaning in life, leisure crafting, and gratitude are predisposing factors for the presence of meaning, based on the framework of meaning-making theory and the meaning maintenance model (Heine et al., 2006; Park, 2010). Researchers have noted that the search for meaning is “the primary motivational force in humans” (Frankl, 1963, p. 121), and “meaning-making attempts following highly stressful events are a near-universal experience” (Park, 2010, p. 282). Meaning in life plays an important role in determining individuals’ well-being. Finding meaning in life is related to low levels of stress (Halama, 2014; Park and Baumeister, 2017) and health-related problems (Steger et al., 2015), as well as high levels of physical and mental health (Czekierda et al., 2017; Yela et al., 2020) and health-promoting behaviors (Brassai et al., 2015).

### Search for Meaning and Presence of Meaning

Meaning in life can be divided into two parts: the search for meaning, referring to a motivational aspect of seeking meaning; and presence of meaning, referring to a belief that one has experienced meaning in one’s present life (Steger et al., 2006). Steger et al. (2008a) empirically tested the relationship between these two variables using the presence-to-search model, which states that the lack of presence of meaning leads to search for meaning; and the search-to-presence model, in which searching for meaning leads to finding meaning. They confirmed a negative relationship between the search for meaning and the presence of meaning, concluding that people search for meaning when they do not experience meaning in their current lives, which was corroborated by follow-up studies (Steger et al., 2009, 2014). Notwithstanding, other studies have yielded conflicting results, demonstrating a positive relationship between the search for meaning and the presence of meaning (Datu, 2016; Chin and Lee, 2020).

This may be attributed to cultural differences. According to Steger et al. (2008b), when individuals reported high levels of the search for meaning, American participants had less presence of meaning, whereas Japanese participants had more presence of meaning. The authors posited that Asians with an interdependent culture have a holistic viewpoint of the world. That is, people in Asian cultures perceive the search for meaning and the presence of meaning to be connected (Yoo and Kim, 2015). Similar results were found in Korean studies (Won et al., 2005; Kim and Lee, 2013). For example, Won et al. (2005) found a significant positive correlation between the search for meaning and the presence of meaning. Moreover, the search for meaning positively influenced the presence of meaning, confirming that the search for meaning was not in itself dysfunctional; rather, it reflected one’s efforts toward self-realization to find meaning in everyday life (Kim and Lee, 2013).

According to the meaning-making theory, individuals under stressful circumstances endeavor to reconcile an appraised meaning of events with a global meaning, which refers to individuals’ general orienting systems (Pargament, 1997). This comprises beliefs, goals, and subjective feelings (Reker and Wong, 1988; Dittman-Kohli and Westerhof, 1999). Specifically, individuals endeavor to find new meaning by changing their perspectives toward stressful situations (Park, 2010; Steger, 2012). Thus, individuals in negative or unfavorable situations in which finding or making meaning is difficult, or their personal meaning systems are threatened, are more likely to discover meaning by searching for meaning, compared to individuals in positive or favorable situations (Chu and Fung, 2021).

Based on the meaning-making theory, we hypothesized that the search for meaning and the presence of meaning will exhibit a positive relationship among ROK Army soldiers. These soldiers are likely to be continuously sensing or experiencing threats to their systems of meaning in life, and, concurrently, they have low levels of presence of meaning. They may be searching for meaning in their daily lives and making efforts to reduce the discrepancy between their appraised meaning and the global meaning. These efforts are expected to foster a higher sense of the presence of meaning, as the meaning-making theory suggests (Park, 2010).

Hypothesis 1: The search for meaning will be positively related to the presence of meaning.

### Leisure Crafting and Meaning in Life

Crafting efforts can be explained using meaning-making processes. Based on the meaning-making theory, the discrepancy between appraised meaning and global meaning that individuals experience in the face of stressful life events is an important driver for individuals to search for meaning to restore damage to their meaning system. By engaging in this deliberate meaning making process, individuals can attain a sense of meaning in life (Park, 2010; Park and George, 2013). Furthermore, the meaning maintenance model suggests that when an individuals’ meaning system is damaged, they try to formulate a new meaning system in another domain where it is relatively easier to build a new meaning system (Heine et al., 2006). That is, individuals under stressful circumstances make efforts to build a meaning system in a new domain to reduce the discrepancy between their appraised meaning and global meaning, and these efforts will lead to a specific crafting behavior. For example, if a person encounters a discrepancy of meaning in their work domain, crafting efforts will be made in non-work domains, such as leisure. Similarly, if a person experiences meaning-related discrepancy in a non-work domain, crafting efforts will be made in the work domain.

De Bloom et al. (2020) demonstrated the overall crafting process (crafting motive, efforts, and outcomes) using the identity-based integrative needs model of crafting. According to this model, the discrepancy between individuals’ psychological needs and reality plays an important role in motivating them to perform, or endeavor to perform, crafting behaviors. When there is a discrepancy between actual and ideal needs, individuals attempt to craft the situation in a way that reduces the discrepancy. Notably, these efforts are made in a proactive, intentional, and deliberate manner, which constitute the main characteristics of crafting. If these efforts are successful in narrowing the discrepancy, individuals can attain various desirable outcomes, such as work-related ones or subjective/psychological well-being (De Boeck et al., 2019). It is also possible that the motive for crafting in one identity domain may influence crafting efforts in the other. For instance, researchers found that unfulfilled needs in the workplace increased individuals’ motive for leisure crafting in non-work domains (Petrou and Bakker, 2016; Petrou et al., 2017). That is, if individual’s needs are not fulfilled in one domain, to compensate for the unsatisfied needs, they may increase their crafting efforts in different domains.

Additionally, leisure crafting is a construct that is differentiated from a simple participation in leisure activities. Petrou and Bakker (2016) defined leisure crafting as the “proactive pursuit of leisure activities targeted at goal setting, human connection, learning and personal development” (p. 2). According to this definition, individuals engaging in leisure crafting actively seek out opportunities to achieve personal growth and development through leisure activities. Moreover, literature on leisure has reported consistently positive relationships between leisure and meaning in life (Newman et al., 2014; Iwasaki et al., 2018). Similarly, research on leisure crafting empirically demonstrates that leisure crafting positively influences work meaning (Lim et al., 2019) and is positively associated with creating meaning in life (Petrou et al., 2017). Berg et al. (2010) were the first to propose the concept of leisure crafting. They qualitatively explained that, by proactively creating leisure time and engaging in leisure crafting, workers could experience meaning that they had not previously experienced in their work domain owing to an unanswered calling. Moreover, individuals who perform tasks involving high stressors might choose to actively engage in leisure crafting, seeking to be compensated for growth, development, and meaning that are unobtainable from their work domain (Petrou and Bakker, 2016).

Applying the findings from these theories to ROK Army soldiers, we predicted that soldiers who experienced loss or a lack of a meaning system in stressful situations during military services would strive to find meaning in their lives. This would be achieved by proactively engaging in activities during their leisure time to seek and restore the meaning system in their military lives. This process would enable them to find the meaning that they lacked during their working hours.

Hypothesis 2: Leisure crafting will mediate the relationship between the search for meaning and the presence of meaning.

### Gratitude and Meaning in Life

To date, gratitude has been defined in a variety of ways, including moral virtue, positive affect, attitude, and life orientation (Wood et al., 2010; Lambert et al., 2012). This study follows the definition by Wood et al. (2010), which describes gratitude as “a part of a wider life orientation toward noticing and appreciating the positive worldwide” (p. 891). Understanding gratitude from the perspective of a comprehensive life orientation is more useful in explaining the various aspects of gratitude. Specifically, gratitude has positive psychological functions and helps in lowering workplace materialism and depression (Lambert et al., 2012; Unanue et al., 2021) and increasing well-being (Jans-Beken et al., 2018; Yoo, 2020). Regarding workers who are frequently exposed to stressful situations, such as soldiers and firefighters, gratitude can function as a protection mechanism. Valikhani et al. (2019) studied a sample of Iranian soldiers and showed that gratitude was negatively associated with stress and poor mental health, and positively correlated with quality of life. Similarly, Lee et al. (2018) found that gratitude is a strong predictor of lowering perceived stress and burnout among firefighters in Korea.

Gratitude is an important element in the framework of meaning-making theory. Specifically, positive reappraisal or reattribution in the meaning-making process plays a critical role in linking gratitude to meaning-making. According to the meaning-making theory (Park, 2010), individuals who experience a discrepancy between their appraised meaning and global meaning after stressful events adopt a meaning-making coping strategy by invoking positive appraisal to reduce distress from the discrepancy.

Thus, cognitive reappraisal or reattribution is closely associated with the construct of gratitude. According to Watkins (2013), gratitude arises through a cognitive process, in which appraisal and attribution are central (Nezlek et al., 2017). If an individual is grateful when they receive benefits, such as presents or assistance from others, gratitude occurs not because of the benefits themselves, but because of the cognitive appraisal and attribution of the benefits. This process is similarly applicable to negative situations. For an individual faced with negative or unfavorable circumstances, a reinterpretation of the situation should precede their feeling of gratitude. Specifically, efforts of cognitive reappraisal to explore positive aspects from negative conditions (that is, the search for meaning) can lead to gratitude.

Gratitude is an important approach for deriving meaning in life. According to the broaden-and-build theory, gratitude broadens individuals’ thought-action repertoires, which subsequently helps build their personal resources by making the cognitive process more flexible (Fredrickson, 2004, 2013). Moreover, gratitude helps people perceive unfavorable situations or events as less negative, thereby reducing their negative influence (Watkins, 2004). Consequently, gratitude helps people find meaning in life by providing cognitive resources with which they can reinterpret negative events or circumstances positively, thus enabling them to view threats as opportunities (Lambert et al., 2009).

Several studies have confirmed a positive relationship between gratitude and meaning in life (i.e., the search for meaning and the presence of meaning). A study on flood survivors indicated that the search for meaning was positively associated with post-traumatic growth (Dursun et al., 2016). Moreover, the authors explained that efforts to find meaning by individuals who have experienced severe pain led to feeling gratitude for their human existence, which subsequently led to post-traumatic growth. In a cross-sectional study conducted with an Israeli sample, gratitude was positively correlated with the search for meaning and the presence of meaning (Russo-Netzer, 2019). Similarly, Datu and Mateo (2015), using a sample of Filipino college students, showed that dispositional gratitude was positively associated with the presence of meaning in life.

Considering the results from studies on gratitude and meaning in life (i.e., the search for meaning and the presence of meaning), we expected that ROK Army soldiers, who are motivated to seek meaning in their lives under difficult and stressful situations, should feel a greater sense of gratitude, which will help them find meaning in their lives.

Hypothesis 3: Gratitude will mediate the relationship between the search for meaning and the presence of meaning.

## Leisure Crafting, Gratitude, and Meaning in Life

Finally, we expected that the search for meaning, leisure crafting, gratitude, and the presence of meaning may have a sequential relationship. Leisure crafting can fulfill humans’ basic psychological needs such as autonomy, competence, and relatedness (Tsaur et al., 2020). That is, individuals’ need for autonomy can be fulfilled by proactively making plans and selecting activities for personal development in their leisure time, and the need for competence can be satisfied by achieving goals related to leisure and acquiring new skills. In addition, the desire for relatedness can be satisfied through human connection and expanding interpersonal relationships (Tsaur et al., 2020). According to De Bloom et al. (2020), individuals experiencing needs discrepancies can fulfill their needs through crafting efforts, which can eventually lead to enhancing the level of their well-being. Army soldiers—who perform tasks that are too simple to experience competence, in an environment with limited autonomy, and are separated from intimate relationships—can meet their basic psychological needs through leisure crafting.

The fulfillment of basic psychological needs can lead to gratitude. Moè and Katz (2020) empirically demonstrated that needs satisfaction of teachers can serve as an emotional resource for reappraisal. Considering that the positive reinterpretation of negative situations is an important facet of gratitude (Watkins, 2013), the fulfillment of basic psychological needs should lead to gratitude through reappraisal. A cross-lagged study by Lee et al. (2015) showed that there exists a positive relationship between fulfillment of basic psychological needs and gratitude.

The above studies suggest that ROK Army soldiers can experience gratitude by engaging in leisure crafting. The ROK Army soldiers work in an environment where fulfillment of basic needs, such as autonomy, competence, and relatedness, is difficult to obtain. However, during leisure time, they can meet their needs by proactively engaging in leisure crafting, which can enable them to experience a higher level of gratitude (Kujanpää et al., 2021). Thus, we formulated a hypothesis that can comprehensively explain the sequential mediation of leisure crafting and gratitude in the relationship between the search for meaning and the presence of meaning.

Hypothesis 4: Leisure crafting and gratitude will sequentially mediate the relationship between the search for meaning and the presence of meaning.

## Materials and Methods

### Participants and Procedure

Participants were ROK army soldiers who were currently on active duty. The surveys were conducted at two different time points over a 2-week interval. To avoid common method bias (Podsakoff et al., 2003), we designed a short-term longitudinal study. Three military units of the ROK army were randomly selected for the survey. After seeking their cooperation, the first survey was conducted on the soldiers who provided informed consent. Prior to our first survey, we explained the study purpose to participants and informed them that survey results would remain confidential. A total of 471 participants responded to the first survey, and 387 of them subsequently engaged in the second survey. Each participant was rewarded a pen in the first survey and a gift worth 2,000 won (approximately 1.7 dollars) in the second survey. Both surveys were conducted face-to-face. Of those who completed the baseline survey, we selected 465 for our final analysis, excluding six participants with insincere responses. There were 381 participants who completed both the baseline and follow-up surveys, indicating an 81.9% retention rate. Although the survey was conducted during the pandemic, the soldiers who participated in the survey were performing their duties normally. We believe that the pandemic did not significantly affect the responses of participants in this study.

All participants were men, given that the mandatory enlisting system in Korea applies only to males, and our survey included only enlisted soldiers. Participants’ ages ranged from 18 to 27 years (*M* = 20.88 years, *SD* = 1.29). Regarding rank, 51% were private first class or below (*n* = 195; Rank 1), 42% were corporals (*n* = 159; Rank 2), and 7% were sergeants (*n* = 27; Rank 3). Regarding religion, 20% were Protestant (*n* = 77), 6% were Catholic (*n* = 24), 6% were Buddhists (*n* = 24), and 68% had no religion (*n* = 256). The time spent on leisure activities ranged from 0 to 43.05 h per week (*M* = 4.61, *SD* = 5.43).

To evaluate whether a systematic pattern occurred in missing data, we performed Little’s (1988) Missing Completely at Random test. The results confirmed that the missing data pattern was random [χ^2^(25) = 26.32, *p* = 0.39]. We also conducted independent samples *t*-tests to determine whether there were differences between the group of participants who completed only the first survey (Time 1 [T1]) and the group who participated both during T1 and T2 (Time 2; the second survey). Our results indicated no differences between the two groups in search for meaning at T1 [*t*(469) = 1.15, *p* = 0.25], leisure crafting [*t*(469) = 1.67, *p* = 0.10], gratitude [*t*(469) = 1.37, *p* = 0.17], and presence of meaning [*t*(469) = 1.14, *p* = 0.25]. This suggests that the attrition was not systematic for the study variables.

At T1, we gathered responses on demographic variables such as age, rank, religion, and average time spent on leisure activities in the past week (for example, running or jogging, playing soccer, watching TV or video clips, playing mobile games, and communicating through social-networking services). We also evaluated study variables, including search for meaning, leisure crafting, gratitude, and presence of meaning. At T2, 2 weeks later, we collected data on presence of meaning.

### Measures

#### Meaning in Life

Meaning in life was measured using the Korean version of the Meaning in Life Questionnaire (Won et al., 2005), which was translated and validated from the original (Steger et al., 2006). The Korean version comprises two subscales: search for meaning and presence of meaning. Search for meaning comprises five items, for example, “I am always looking to find my life’s purpose” and “I am seeking a purpose or mission for my life.” Presence of meaning comprises five items, for example, “I understand my life’s meaning” and “I have a good sense of what makes my life meaningful.” Participants responded using a 7-point Likert scale (1 = absolutely untrue, 7 = absolutely true). Won et al. (2005) reported Cronbach’s an α coefficient of 0.88 each for search for meaning and presence of meaning. In our study, Cronbach’s αs were 0.91 and 0.83, respectively.

#### Leisure Crafting

To measure the levels of leisure crafting, we utilized the translated version of the Leisure Crafting Scale (Petrou and Bakker, 2016). The original English version was translated into Korean by the authors, who are fluent in English and Korean. A native English speaker, who is bilingual in Korean, verified correspondence with the original using back-translation. This scale comprises a single factor containing nine items, for example, “I try to increase my skills through leisure activities” and “My leisure time is a chance for me to grow and develop.” Responses are measured using a 5-point Likert scale (1 = not at all, 5 = very much). Petrou and Bakker (2016) reported a Cronbach’s α of 0.92, and in the current study, it was 0.93.

#### Gratitude

Gratitude was measured using the K-GQ-6 Scale—the Korean-validated version (Kwon et al., 2006) of the Gratitude Questionnaire (McCullough et al., 2002). This scale comprises a single factor with six items (two items are reverse scored). For example, “I have so much in life to be thankful for” and “I am grateful to a wide variety of people.” The items measure the intensity, frequency, range, and density of gratitude. Participants responded using a 7-point Likert scale (1 = strongly disagree, 7 = strongly agree). Cronbach’s αs were 0.82 in the original version, 0.85 in the Korean-validated version, and 0.88 in this study.

#### Control Variables

In our analyses, we controlled for rank and religion, both of which showed correlations with other variables, and the average leisure time for the past 1 week. Furthermore, we controlled for presence of meaning at T1 to observe the change over time.

### Data Analyses

First, we used IBM SPSS v. 21 to estimate the means, standard deviations, internal consistency coefficients, and correlations between variables. Then, we conducted confirmatory factor analyses to test the measurement model, and structural equation modeling to test the research hypotheses using IBM SPSS AMOS 18.0. Item parceling is a useful tool to enhance model fit when we use the structural equation modeling (MacCallum et al., 1999). Thus, we created item parcels using the observed variables of leisure crafting and gratitude in the measurement and structural model analyses. Because our study had a two-wave longitudinal design, there were participants who responded only once out of two surveys. When analyzing missing data, many researchers recommend using a full information maximum likelihood (FIML) method that uses information from all observed variable data to obtain more unbiased outcomes (Arbuckle, 1996; Yuan and Bentler, 2000). Accordingly, we adopted the FIML method in this study. To test indirect effects, we used the Monte Carlo approach (Preacher and Selig, 2012), and we set the bias-corrected confidence interval (CI) to 95% (Taylor et al., 2008). The indirect effects are interpreted as significant when the 95% CI does not include zero (Shrout and Bolger, 2002).

## Results

### Descriptive Statistics and Correlations Between Variables

Table 1 shows the means, standard deviations, internal consistency reliability, and the correlation coefficients derived from observed total scores. Internal consistency was confirmed, as Cronbach’s αs ranged between 0.83 and 0.93. All correlations between variables were consistent with our predicted direction. Search for meaning was positively correlated with leisure crafting (*r* = 0.64, *p* < 0.001), gratitude (*r* = 0.52, *p* < 0.001), and presence of meaning (*r* = 0.45, *p* < 0.001). Leisure crafting was positively correlated with gratitude (*r* = 0.49, *p* < 001) and presence of meaning (*r* = 0.41, *p* < 0.001). Gratitude was positively correlated with presence of meaning (*r* = 0.48, *p* < 0.001).

**TABLE 1:** Means, standard deviations, correlations, and Cronbach’s alphas of the study variables (*N* = 465).

		*M*	*SD*	1	2	3	4	5	6	7	8	9	10	11
1	Age	20.88	1.29	–										
2	Rank 1	0.37	0.48	−0.11*	–									
3	Rank 2	0.42	0.49	0.17**	−0.65***	–								
4	Rank 3	0.07	0.26	0.20***	−0.21***	−0.23***	–							
5	Religion	0.33	0.47	–0.03	0.04	0.02	–0.04	–						
6	Leisure time	4.61	5.43	–0.02	0.01	0.03	–0.04	–0.02	–					
7	SM (T1)	5.46	1.12	0.09	−0.11*	0.10	0.02	0.07	0.03	(0.91)				
8	LC (T1)	3.85	0.77	0.07	–0.09	0.04	0.07	0.10	0.04	0.64***	(0.93)			
9	GR (T1)	5.46	1.09	0.06	–0.05	0.01	0.12*	0.11*	–0.03	0.52***	0.49***	(0.88)		
10	PM (T1)	5.01	1.27	0.09	–0.06	–0.01	0.08	0.09	–0.03	0.59***	0.53***	0.52***	(0.86)	
11	PM (T2)	4.88	1.20	0.10	–0.08	0.03	0.05	0.18***	–0.05	0.45***	0.41***	0.48***	0.69***	(0.83)

Rank 1 (0 = private, 1 = private first class), Rank 2 (0 = private, 1 = corporal), Rank 3 (0 = private, 1 = sergeant); Religion (0 = non, 1 = have); Numbers in parentheses are reliability coefficients. M, mean; SD, standard deviation; SM, search for meaning; LC, leisure crafting; GR, gratitude; PM, presence of meaning. *p < 0.05, **p < 0.01, ***p < 0.001.

### Measurement Model Analysis

Before conducting primary analysis on the structural model, we tested the measurement model through a confirmatory factor analysis to ensure that the observed variables provided satisfactory explanations for the latent variables. Therefore, we conducted item parceling on the indicators of leisure crafting and gratitude. Specifically, the nine items from leisure crafting were grouped into three parcels, each with three items in order. The six items from gratitude were grouped into two parcels, each with three items in order. When we evaluated whether indicators were loaded well into the latent variables, all indicator values were 0.72 or higher, except for presence of meaning, which was reverse coded. This confirmed the strong relationship between the latent and observed variables. The fit indices of the measurement model were verified using observed variables whose factor loadings were significant. The results showed that model fit indices of the measurement model were acceptable [χ*^2^*(71) = 244.58, *p* < 0.001; CFI = 0.97; TLI = 0.95; RMSEA = 0.07; AIC = 340.58; Hu and Bentler, 1999].

### Structural Model Analysis

**FIGURE 1:**
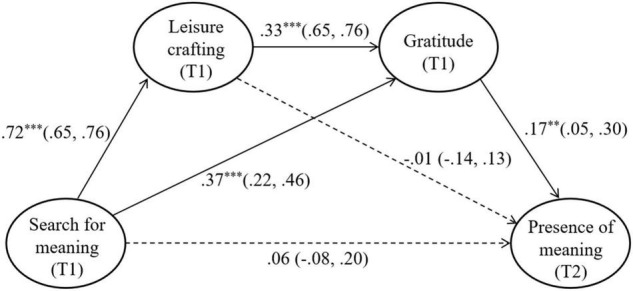
Final structural model with standardized coefficients (95% confidence intervals) of direct effects. ^**^p < 0.01, ^***^p < 0.001.

We conducted a structural equation modeling analysis to test our hypotheses. Figure 1 shows the final structural model with standardized coefficients (95% confidence intervals) of direct effects. Existing research on the relationship between search for meaning and presence of meaning did not produce consistent results (Steger and Kashdan, 2007; Steger et al., 2014). Thus, the “search for meaning → presence of meaning” path was deleted in our full mediation model. Next, the model fit of the partial mediation model, which set the relationships between all latent variables based on our hypotheses, was compared with that of the full mediation model. As shown in Table 2, the differences between the two models were not significant [Δχ^2^(1) = 0.64, *n.s.*], and the partial mediation model was selected as the final model [χ^2^(136) = 390.93, *p* < 0.001; CFI = 0.96; TLI = 0.94; RMSEA = 0.06; AIC = 578.93].

**TABLE 2:** Results of the structural model fit comparison analysis.

	χ*^2^*	*df*	χ*^2^/df*	CFI	TLI	RMSEA	AIC
Partial mediation model	390.93***	136	2.88	0.96	0.94	0.06	578.93
Full mediation model	391.57***	137	2.86	0.96	0.94	0.06	577.57

N = 465; ***p < 0.001. CFI, Comparative Fit Index; TLI, Tucker-Lewis Index; RMSEA, Root Mean Square Error of Approximation; AIC, Akaike Information Criteria.

To assess the partial mediation model, we first tested the relationship between search for meaning and presence of meaning without the mediators (total effect). The results showed that the total effect was significant (β = 0.13, *p* < 0.05), and therefore, Hypothesis 1 was supported.

As the next step, we tested the indirect effects of leisure crafting and gratitude between search for meaning and presence of meaning, by calculating 95% bias-corrected CIs using a Monte Carlo approach (Preacher and Selig, 2012). The results indicated that gratitude mediated the relationship between search for meaning and presence of meaning (β = 0.06, 95% CI [0.01, 0.11]), and that leisure crafting and gratitude sequentially mediated the relationship between search for meaning and presence of meaning (β = 0.04, 95% CI [0.01, 0.08]). Therefore, Hypotheses 3 and 4 were supported. However, Hypothesis 2 was not supported as the relationship between leisure crafting and presence of meaning turned out to be non-significant (β = –0.01, 95% CI [–0.10, 0.10]).

Finally, we calculated the percent mediation (i.e., indirect effect/total effect) to compare the indirect effect with the total effect. The percent mediation was 0.77, suggesting that the indirect effect represented 77% of the total effect.

## Discussion

The purpose of this study was to identify the mechanisms behind the relationship between search for meaning and presence of meaning, with an emphasis on the mediating role of leisure crafting and gratitude. We conducted two-wave surveys with a 2-week interval using a sample of ROK Army soldiers. Findings revealed mediating roles of leisure crafting and gratitude in the relationship between the search for meaning and the presence of meaning.

### Theoretical Implications

Our study findings have several theoretical implications. First, our research findings supported the search-to-presence model, which assumes that searching for meaning leads to presence of meaning (Steger et al., 2008a). To date, studies on the relationship between search for meaning and presence of meaning have reported mixed results. For example, the relationship between the two was generally negative in Western cultures, and generally positive in Eastern cultures (Steger et al., 2008b; To, 2016; Chin and Lee, 2020). The correlation between the search for meaning and presence of meaning was 0.45 in this study, which is within the range of correlation coefficient values identified in other studies conducted in interdependent cultures. For example, in a study of Japanese young adults and Korean college students, the correlation between the two variables was 0.24 and 0.28, respectively (Steger et al., 2008b; Kim and Lee, 2013). In other studies conducted in Korea, it was higher at 0.52 (Won et al., 2005; Chin and Lee, 2020). This study provides evidence that in Eastern cultures, the search-to-presence model, where seeking meaning leads to discovery, is more applicable.

Our results corroborate extant studies asserting that individuals living in Eastern cultures tend to regard the world from a more holistic perspective, compared to those in Western cultures, perceiving the search for meaning and the presence of meaning to be connected (Steger et al., 2008b; Yoo and Kim, 2015). That is, they do not regard the search for meaning as dysfunctional but accept it as a part of their efforts to find meaning in life (Kim and Lee, 2013). Therefore, our finding that the total effect of search for meaning on presence of meaning is significant provides evidence that in Eastern cultures, the search-to presence model, where seeking meaning leads to discovery, is more applicable (Datu, 2016; Chu and Fung, 2021).

However, our results revealed that the search for meaning and presence of meaning are not directly related. This may be because our study is longitudinal, while other studies demonstrating a significant direct relationship between the search for meaning and presence of meaning were cross-sectional (Yoo and Kim, 2015; Chin and Lee, 2020). Another possibility is that the relationship between the search for meaning and presence of meaning was explained mostly by two mediators such as leisure crafting and gratitude. In fact, the percent mediation of 0.77 suggests that the indirect effect of mediators accounted for 77% of the total effect of search for meaning on presence of meaning.

Second, we confirmed the role of gratitude in the mechanism through which the search for meaning led to the presence of meaning. Individuals who seek meaning tend to exhibit more gratitude, which in turn facilitates finding meaning in their lives. Specifically, individuals in stressful situations endeavor to find meaning in their lives, and these efforts accompany a reappraisal of the negative events or circumstances surrounding them (Park, 2010). Considering that gratitude stems from a cognitive process in which individuals recognize external factors (Watkins, 2013), individuals seeking meaning can experience gratitude by reappraising negative situations. Gratitude expands individuals’ thought–action repertoires, thereby contributing to enriching cognitive resources. Moreover, it causes people to reinterpret negative events or circumstances more positively, enabling them to find meaning in life (Fredrickson, 2004, 2013). From this perspective, participants in our study likely experienced gratitude when they were searching for meaning, which subsequently led them to find meaning in life.

Third, we expanded the boundaries of research in this area by confirming the indirect effects of gratitude in the relationship between the search for meaning and presence of meaning. Previous research on individuals in unfavorable conditions such as natural disasters (e.g., survivors of flooding) showed that their efforts to search for meaning were associated with post-traumatic growth (Dursun et al., 2016). Moreover, those who experience growth after traumatic experiences tend to exhibit character strengths such as a greater appreciation of life, including gratitude (Tedeschi and Calhoun, 1995; Peterson et al., 2008). Our findings empirically tested the meaning-making theory (Park, 2010) and the broaden-and-build theory (Fredrickson, 2004, 2013). Therefore, our study is consistent with extant research that tested the relationships between the search for meaning, gratitude, and the presence of meaning (Dursun et al., 2016; Liao and Weng, 2018; Russo-Netzer, 2019). Our study is significant in that it has proved the value of gratitude in the relationship between search for meaning and presence of meaning among ROK Army soldiers who serve under stressful circumstances.

Last, our results are the first to confirm the sequential mediation effects of leisure crafting and gratitude in the relationship between the search for meaning and presence of meaning. Human beings are motivated to seek meaning. Therefore, when their system of meaning is threatened, they attempt to construct new systems of meaning in domains where finding meaning is easier (Frankl, 1963; Heine et al., 2006).

The integrative crafting model states that individuals who experience a discrepancy of needs in one domain, attempt crafting in other domains to narrow the discrepancies they experience (De Bloom et al., 2020). From this perspective, our findings suggest that ROK Army soldiers, who experience discrepancies between their appraised meaning and global meaning under stressful circumstances, seek to apply crafting in their leisure time rather than in their work to reduce discrepancies in their meaning system.

Specifically, a direct relationship between leisure crafting and the presence of meaning was not significant in our study. Thus, indirect effects could not be confirmed for the “search for meaning → leisure crafting → presence of meaning” path. However, the indirect effect was statistically significant for the “search for meaning → leisure crafting → gratitude → presence of meaning” path. This implies that it is difficult for ROK Army soldiers to find meaning by simply engaging in leisure crafting during their leisure time. Thus, gratitude, which can be acquired from leisure crafting, is a prerequisite for them to find meaning in life.

When basic psychological needs such as autonomy, competence, and relatedness are unmet, individuals seek to fulfill their needs through crafting behaviors, such as leisure crafting (De Bloom et al., 2020). Moreover, fulfilled psychological needs are then used as a psychological resource for reappraisal. Reappraisal of negative situations or environments is a major feature of gratitude (Watkins, 2013). Therefore, it can be inferred that leisure crafting can lead to gratitude by fulfilling the basic psychological needs of individuals. From this perspective, the significant sequential indirect effect of leisure crafting and gratitude found in our study expands the boundaries of research on leisure crafting and gratitude.

### Limitations and Future Research Directions

Despite our study strengths, our results had several limitations. First, we selected a 2-week interval, two-wave study to overcome the limitations of a cross-sectional study. However, a 2-week duration may be insufficient, considering the stable nature of the meaning in life constructs (Steger and Kashdan, 2007). Furthermore, we utilized data measured at two different time points—with predictors and mediators measured at T1, and criterion variables measured at T2. Three variables—the search for meaning, leisure crafting, and gratitude—were all measured in T1, making it difficult to verify temporal precedence between these variables. Future studies should employ more frequent measurement times with longer intervals. For example, four-wave data points would be ideal to verify the sequential mediation model established in this study.

Second, in our research, the constructs of leisure crafting and gratitude were assumed and measured as a single factor. Subsequent studies should utilize multidimensional measurements of leisure crafting and gratitude, so that diverse aspects of these constructs can be captured (Card, 2019; Tsaur et al., 2020).

Third, participants were ROK Army soldiers, who have an Eastern cultural background. This limits the generalizability of our findings. Replicating our study using diverse cultural and occupational groups with stressful working conditions such as police, firefighters, and nurses may prove useful.

Finally, to circumvent common method bias, we conducted a short-term longitudinal study. However, there remains the limitation that our data were collected from the same sources. Although we collected data at two time points to mitigate the potential impact of common method variance, the use of data from a single source may introduce the possibility of common method bias. Future research should use data from multiple sources, such as superiors or colleagues, as well as self-reports, to reduce the problems associated with common method bias (Podsakoff et al., 2003).

## Conclusion and Practical Implications

The presence of meaning is not acquired naturally; rather, it is developed through effort (Chu and Fung, 2021). Therefore, individuals should strive to actively find meaning (Park, 2010; Yang et al., 2019). If an individual encounters deficiency of meaning in the work domain, they attempt to pursue activities in non-work domains to seek meaning (Heine et al., 2006; De Bloom et al., 2020). Existing research suggests that the search for meaning is more effective for individuals in strenuous circumstances (for example, physical diseases, financial difficulties, and low level of presence of meaning) to find meaning (To, 2016; Szcześniak et al., 2020; Chu and Fung, 2021). Therefore, efforts to search for meaning can be made through intentional and proactive activities. Such efforts facilitate people to feel gratitude through reappraisal by fulfilling their basic psychological needs.

Soldiers serving in the ROK Army find themselves in a high-stress environment and struggle to find meaning from their duties apart from patriotism. Moreover, their system of meaning can be easily infringed upon owing to mandatory (or involuntary) enlistment, a strict hierarchical culture, and repetition of simple tasks. Our findings suggest that it is possible to feel a sense of meaning if individuals strive to seek meaning in life, proactively engage in leisure crafting, and experience a sense of gratitude, even under adverse circumstances. Therefore, making efforts to find meaning in unfavorable conditions by engaging in leisure crafting and experiencing a sense of gratitude can be an important driving force for individuals to attain a sense of presence of meaning. Meaning in life, which Army soldiers of ROK can discover and experience through the above-mentioned processes, should play a vital role in lowering their stress levels and enhancing their mental health.

Our findings have practical implications for military supervisors. Considering the positive aspects of gratitude experienced in organizations (Di Fabio et al., 2017), the military should provide opportunities for soldiers to proactively participate in leisure activities related to meaning (e.g., leisure crafting). This is because leisure crafting helps soldiers experience higher levels of gratitude, thereby helping them find meaning in situations that are generally considered unfavorable. Particularly, the military should endeavor to guarantee an environment that helps soldiers to proactively utilize their leisure time. For soldiers, who are often required to have controlled lifestyles, leisure time is the only time where their autonomy is guaranteed. Institutional and systematic support to facilitate soldiers’ engagement in leisure crafting activities should be beneficial for enhancing their psychological health.

Military supervisors can conduct campaigns to perform gratitude practices, highlighting the benefits of being grateful. Simple interventions can lead to positive results regarding gratitude (Cheng et al., 2015). Thus, the military can engage in gratitude interventions such as writing gratitude diaries, sending thank you letters and messages, and counting blessings, to help soldiers experience their meaning in life (Valikhani et al., 2019). Practicing gratitude, which facilitates discovering one’s meaning in life, can have pragmatic implications for improving the mental health of soldiers. The mental health practitioners who provide counseling or consulting services for soldiers with low levels of meaning in life can motivate and encourage soldiers to engage in various daily gratitude practices to regain or enhance their sense of meaning in life.

## Data Availability Statement

The datasets presented in this article are not readily available because the release of the raw data should be approved by the Ministry of National Defense and the South Korea Army Headquarters. Requests to access the datasets should be directed to JL, lji4019@yonsei.ac.kr.

## Ethics Statement

The studies involving human participants were reviewed and approved by the Yonsei University Institutional Review Board. The patients/participants provided their written informed consent to participate in this study.

## Author Contributions

JL collected the data, analyzed the model, and led the drafting of the manuscript as a first author. JY and YS participated in the rewriting of the manuscript and consulted on the research design. YS supervised the process of this work and he repeatedly revised the manuscripts. All authors provided critical feedback, approved the final version of the manuscript, and designed the study.

## Conflict of Interest

The authors declare that the research was conducted in the absence of any commercial or financial relationships that could be construed as a potential conflict of interest.

## Publisher’s Note

All claims expressed in this article are solely those of the authors and do not necessarily represent those of their affiliated organizations, or those of the publisher, the editors and the reviewers. Any product that may be evaluated in this article, or claim that may be made by its manufacturer, is not guaranteed or endorsed by the publisher.
